# Fire at the New Veterinary College, Edinburgh

**Published:** 1884-04

**Authors:** 


FIRE AT THE NEW VETERINARY COLLEGE, EDINBURGH.—The New Veterinary College recently opened was partially destroyed by fire on the night of January 26th. The loss was estimated at £1,000, which is covered by insurance. A more serious loss was that of the library of the Edinburgh Veterinary Medical

Society, which was totally destroyed. It was founded by the late Professor Dick in 1834.

